# Cyclic tensile force modifies calvarial osteoblast function via the interplay between ERK1/2 and STAT3

**DOI:** 10.1186/s12860-023-00471-8

**Published:** 2023-03-08

**Authors:** Xiaoyue Xiao, Shujuan Zou, Jianwei Chen

**Affiliations:** 1 State Key Laboratory of Oral Diseases and National Clinical Research Center for Oral Diseases, Department of Orthodontics, West China Hospital of Stomatology, Sichuan University, Chengdu, China; 2 Chongqing Key Laboratory of Oral Disease and Biomedical Sciences, Stomatological Hospital of Chongqing Medical University, Chongqing, China; 3 Chongqing Municipal Key Laboratory of Oral Biomedical Engineering of Higher Education, Stomatological Hospital of Chongqing Medical University, Chongqing, China

**Keywords:** Osteoblasts, Cyclic tensile stress, Osteogenesis, ERK1/2, STAT3

## Abstract

**Background:**

Mechanical therapies, such as distraction osteogenesis, are widely used in dental clinics. During this process, the mechanisms by which tensile force triggers bone formation remain of interest. Herein, we investigated the influence of cyclic tensile stress on osteoblasts and identified the involvement of ERK1/2 and STAT3.

**Materials and methods:**

Rat clavarial osteoblasts were subjected to tensile loading (10% elongation, 0.5 Hz) for different time periods. RNA and protein levels of osteogenic markers were determined using qPCR and western blot after inhibition of ERK1/2 and STAT3. ALP activity and ARS staining revealed osteoblast mineralization capacity. The interaction between ERK1/2 and STAT3 was investigated by immunofluorescence, western blot, and Co-IP.

**Results:**

The results showed that tensile loading significantly promoted osteogenesis-related genes, proteins and mineralized nodules. In loading-induced osteoblasts, inhibition of ERK1/2 or STAT3 decreased osteogenesis-related biomarkers significantly. Moreover, ERK1/2 inhibition suppressed STAT3 phosphorylation, and STAT3 inhibition disrupted the nuclear translocation of pERK1/2 induced by tensile loading. In the non-loading environment, inhibition of ERK1/2 hindered osteoblast differentiation and mineralization, while STAT3 phosphorylation was elevated after ERK1/2 inhibition. STAT3 inhibition also increased ERK1/2 phosphorylation, but did not significantly affect osteogenesis-related factors.

**Conclusion:**

Taken together, these data suggested that ERK1/2 and STAT3 interacted in osteoblasts. ERK1/2-STAT3 were sequentially activated by tensile force loading, and both affected osteogenesis during the process.

**Supplementary Information:**

The online version contains supplementary material available at 10.1186/s12860-023-00471-8.

## Introduction

Mechanical therapies have emerged as common choices in dental clinics, such as distraction osteogenesis (DO), orthodontic tooth movement, and maxillary advancement/expansion. It is well known that tensile force stimulates osteogenic activity, thus driving bone formation. In recent years, researchers have been paying increasing attention to mechanotransduction, by which mechanical force is translated into biochemical signals and thereby initiates osteogenesis.

Osteoblasts, which are derived from mesenchymal stem cells, are the main bone-forming cells in response to mechanical loading [1]. It has been reported that osteoblasts are involved in bone formation from the synthesis of matrix proteins to mineralization [2, 3]. In addition, osteoblastic cells such as MC3T3-E1 display mechanosensitive characteristics [4] and their function influences tension-induced osteogenesis [5]. Therefore, it is of critical importance to clarify mechanotransduction in osteoblasts, which could help develop mechanism-based adjuvant methods in favor of bone formation.

Extracellular signal-regulated kinase 1/2 (ERK1/2) and signal transducer and activator of transcription 3 (STAT3) are two key mechanosensory molecules. ERK1/2 reportedly mediates the conversion of external mechanical stimulation to intracellular biochemical signals [6, 7] and then triggers cellular response. The mechanosensitive properties of STAT3 have also been confirmed in previous studies. Its deletion impairs load-driven bone formation in long bones [8], as well as bone remodeling during orthodontic tooth movements [5].

A number of biological processes have been proposed to involve crosstalk between signaling pathways. ERK1/2 and STAT3 may coordinate in various ways to modulate physiological processes. In mechanical loading-responsive myocardial cells, ERK1/2 and STAT3 were the downstream effectors of mechanical signal transmission [9]. Crosstalk between ERK1/2 and STAT3 plays a critical role in osteogenic lineage cells as evidenced by a growing body of research. Hyperactivation of ERK1/2 following STAT3 deletion enhances the proliferation ability of bone mesenchymal stem cells (BMSCs) exposed to granulocyte colony-stimulating factor [10, 11]. Additionally, in MC3T3-E1 treated with IL-6/IL-6 soluble receptor, ERK1/2 and STAT3 played antagonistic actions in MC3T3-E1 differentiation and the inhibition of STAT3 activated ERK1/2 [12]. It should be noted that there is no conclusive interaction between the two signaling pathways, and they could counteract or compensate for each other to coordinate biological events. Thus, we speculate that crosstalk between ERK1/2 and STAT3 exists in osteoblasts under mechanical loading and contributes to the osteogenic response.

In this study, we first examined the effects of cyclic tensile stress on osteogenesis-related factors in rat primary osteoblasts. Next, inhibition of ERK1/2 or STAT3 was carried out on osteoblasts to determine their roles and potential interdependences.

## Results

### Osteogenesis-related factors were upregulated by cyclic tensile loading in a time-dependent manner

**Fig. 1:**
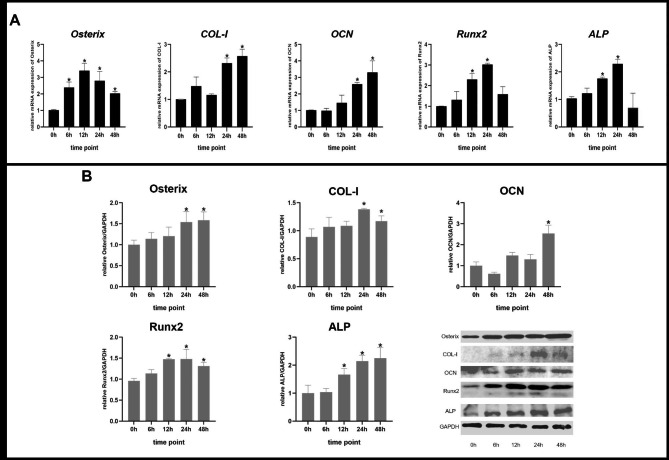
The osteogenic gene and protein expression of primary osteoblasts under mechanical tensile loading for 6, 12, 24, and 48 h. (A) The relative gene expressions of *Osterix, COL-I, OCN, Runx2, and ALP* were increased over time. (B) The protein levels of Osterix, COL-I, OCN, Runx2, and ALP were determined by western blots and their densitometric analysis. Bars represent mean ± SD, *p < 0.05, compared with the control group (0 h group)

Cyclic tensile loading (10% elongation, 0.5 Hz) was imposed on osteoblasts using the Flexcell tension system for 6, 12, 24, and 48 h. Osteoblasts not subjected to load served as the control group. To investigate the effects of tensile loading on osteogenesis in osteoblasts, the expression of osteogenesis-related genes was examined by qPCR and western blots. Compared with the control group, mRNA levels of Osterix, COL-I, OCN, Runx2, and ALP demonstrated varying degrees of increase after strain load. Osterix mRNA level was significantly upregulated as early as 6 h of strain loading. Runx2 and ALP mRNA expression peaked at 24 h and then decreased at 48 h. COL-I and OCN mRNA expression increased over time, peaking at 48 h (Fig. 1A). Figure 1B showed that their protein expression changes were in agreement with the changes in the corresponding mRNA.

### ERK1/2 and STAT3 were phosphorylated and translocated to the nucleus in response to tensile loading

**Fig. 2:**
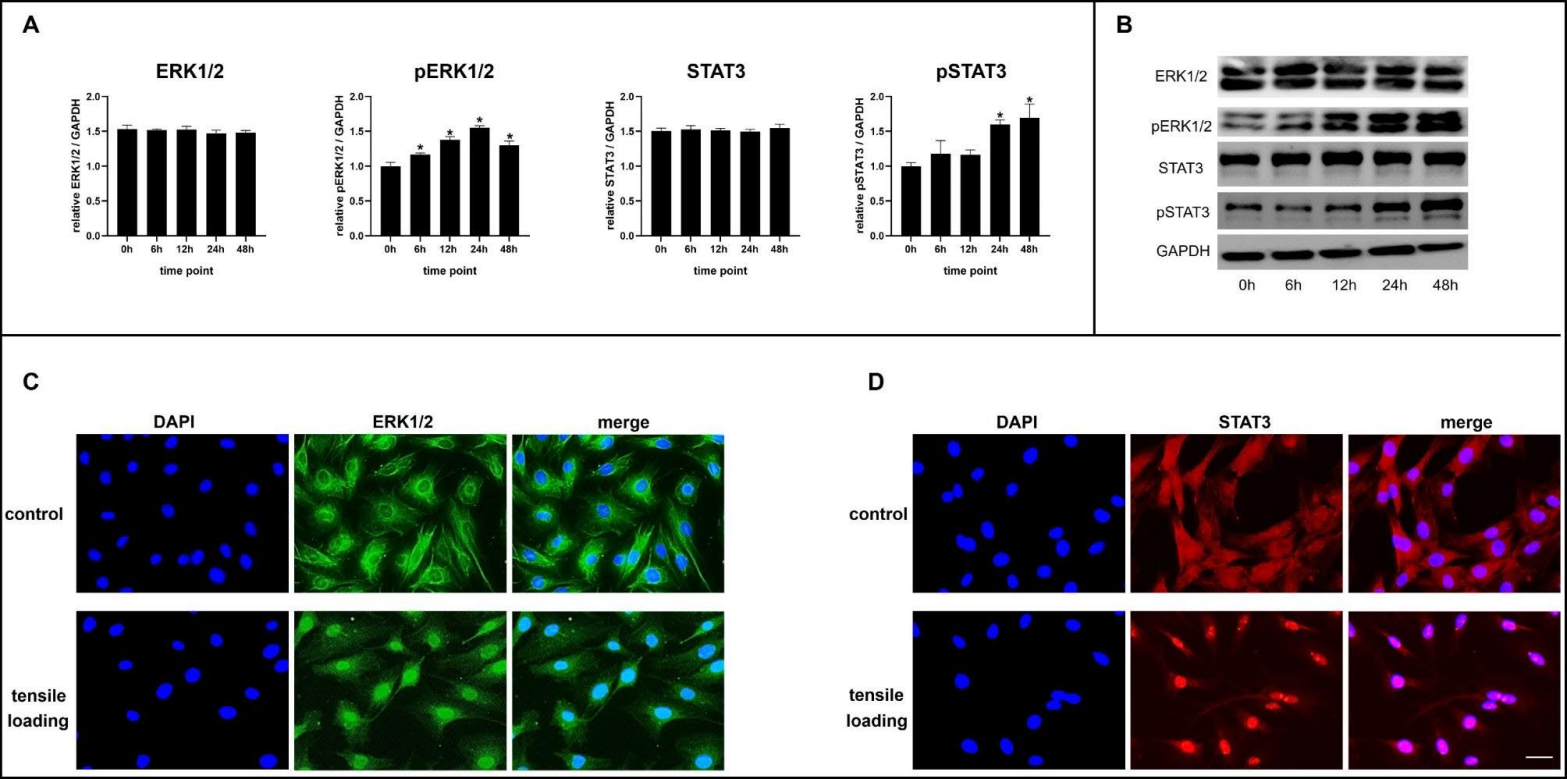
ERK1/2 and STAT3 were activated after mechanical tensile loading. (**A)** Densitometric analysis of ERK1/2, pERK1/2, STAT3, and pSTAT3 protein levels in osteoblasts under tensile loading for 6, 12, 24, and 48 h. **(B)** Representative images of ERK1/2, pERK1/2, STAT3, and pSTAT3 protein changes under tensile loading for 6, 12, 24, and 48 h. **(C-D)** Immunofluorescence assay showed nuclear translocation of ERK1/2 and STAT3 protein induced by tensile loading for 24 h. Scale bar: 25 μm, Bars represent mean ± SD. *p < 0.05, compared with the control group (0 h group)

We analyzed the protein levels of ERK1/2, STAT3, and their phosphorylation after different time periods of loading using western blots. ERK1/2 and STAT3 protein expression was not significantly altered overall. However, the protein level of pERK1/2 was greatly increased at 6 h of strain loading and reached its maximum at 24 h. After 24 h of mechanical loading, pSTAT3 expression increased significantly, followed by further increases after 48 h (Fig. 2A and B).

Immunofluorescence was used to determine the subcellular localization of ERK1/2 and STAT3. As shown in Fig. 2C, ERK1/2 protein in the control group was mainly located in the cytoplasm, whereas 24 h of tensile stress loading induced ERK1/2 into the nucleus (Fig. 2C). STAT3 protein was found in both the cytoplasm and nucleus in the control group. The accumulation of STAT3 in the nucleus was also observed after 24 h of loading (Fig. 2D).

### ERK1/2 inhibition suppressed osteoblast differentiation- and mineralization-related factors

**Fig. 3:**
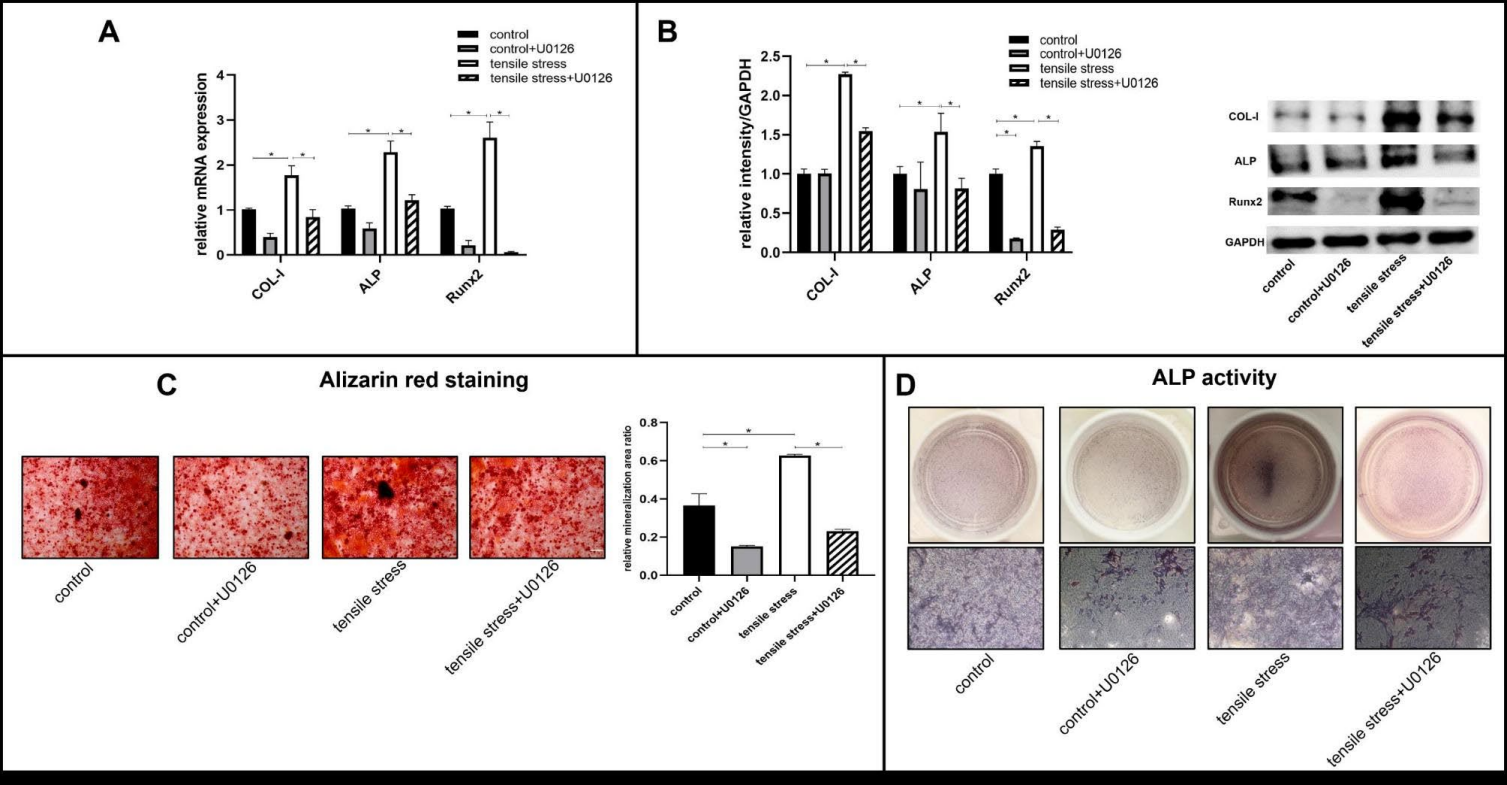
The osteoblast differentiation and mineralization of primary osteoblasts after ERK1/2 inhibition in the presence or absence of tensile loading. Osteoblasts were treated with U0126 for 12 h, followed by mechanical loading of 24 h. (A) The mRNA expressions of *COL-I, ALP, and Runx2* were detected by qPCR. (B) Western blots analysis of the osteogenic markers COL-I, ALP, and Runx2. (C) Alizarin red staining showed the formation of mineralized nodules of osteoblasts after ERK1/2 inhibition and (D) ALP activity (scale bar: 200 μm). Bars represent mean ± SD. *p < 0.05

To assess ERK1/2 involvement in osteogenesis, we incubated the cells with the ERK1/2 inhibitor U0126 (10µM) for 12 h, followed by 24 h of mechanical loading. The mRNA levels of osteogenesis-related factors were measured using qPCR. There was a significant increase in COL-I, ALP, and Runx2 mRNA expression after tensile stress loading compared to the control group. It was observed that inhibition of ERK1/2 greatly reduced the expressions of COL-I, ALP, and Runx2 regardless of loading presence (Fig. 3A). Consistent with mRNA alteration, protein expressions of COL-I, ALP, and Runx2 were markedly increased after loading, but were reversed by ERK1/2 inhibition. In the control + U0126 group, Runx2 protein level was significantly reduced, whereas that of COL-I and ALP was not affected (Fig. 3B).

The mineralization capacity was further examined by ALP and ARS staining. As illustrated in Fig. 3C, alizarin red staining revealed that more mineralized nodules were observed after loading, but they were reduced when ERK1/2 was inhibited. The formation of mineralized nodules in the control + U0126 group was less pronounced than in the control group. According to Fig. 3D, ALP activity was enhanced by tensile stress, while ERK1/2 inhibition reduced its intensity.

### STAT3 regulated the promoting effects of mechanical strain on osteoblast differentiation and mineralization

**Fig. 4:**
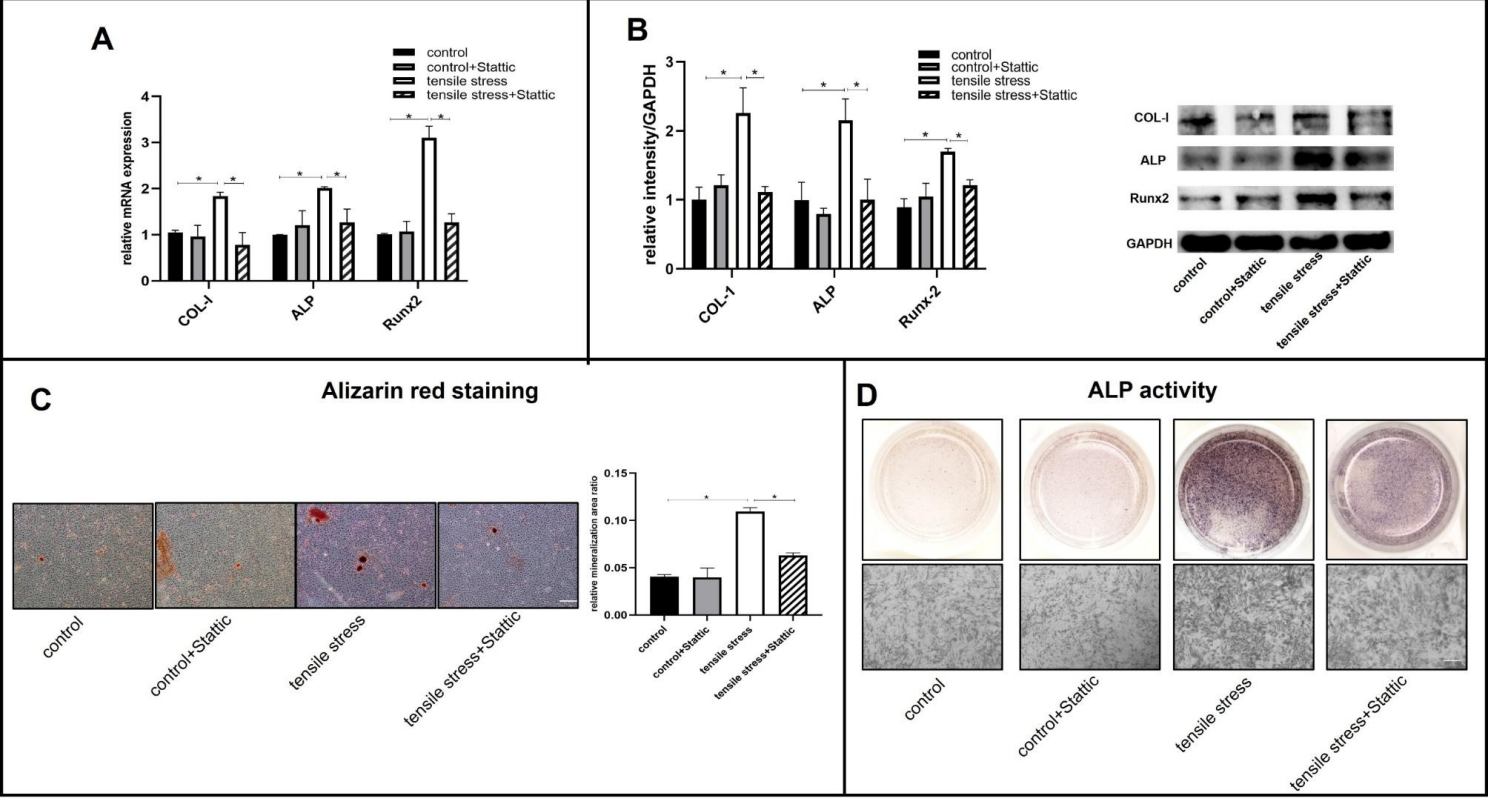
The osteoblast differentiation and mineralization of primary osteoblasts after STAT3 inhibition in the presence or absence of tensile loading. Osteoblasts were treated with Stattic for 6 h, followed by mechanical loading for 24 h (A) The mRNA expressions of *COL-I, ALP, and Runx2* were detected by qPCR. (B) Western blots analysis of the osteogenic markers COL-I, ALP, and Runx2. (C) Alizarin red staining showed the formation of mineralized nodules of osteoblasts after STAT3 inhibition and (D) ALP activity (scale bar: 200 μm). Bars represent mean ± SD. *p < 0.05

To assess STAT3 involvement in osteogenesis, we treated the cells with STAT3 inhibitor Stattic (5µM) for 6 h, followed by 24 h of loading. There was no significant difference between the control group and the control + Stattic group in terms of the mRNA levels of COL-I, ALP, and Runx2. In response to mechanical loading, mRNA expression of COL-I, ALP, and Runx2 increased, but STAT3 inhibition dramatically reduced it (Fig. 4A). As shown by western blot analysis, Runx2, ALP, and COL-I protein expression did not differ significantly between the control group and the control + Stattic group. STAT3 inhibition, however, attenuated their increased expression induced by mechanical loading (Fig. 4B).

ARS and ALP staining were also performed to identify mineralization capacity. Tensile stress loading greatly enhanced ALP staining intensity and mineralized nodule formation. In the tensile stress + Stattic group, ALP activity and mineralized nodule formation were significantly decreased compared with those in the tensile stress group. It was noteworthy that STAT3 inhibition did not significantly impact ALP staining and mineralized nodule formation in the control + Stattic group (Fig. 4C and D).

### ERK1/2 and STAT3 had interaction and their activation influenced each other

**Fig. 5:**
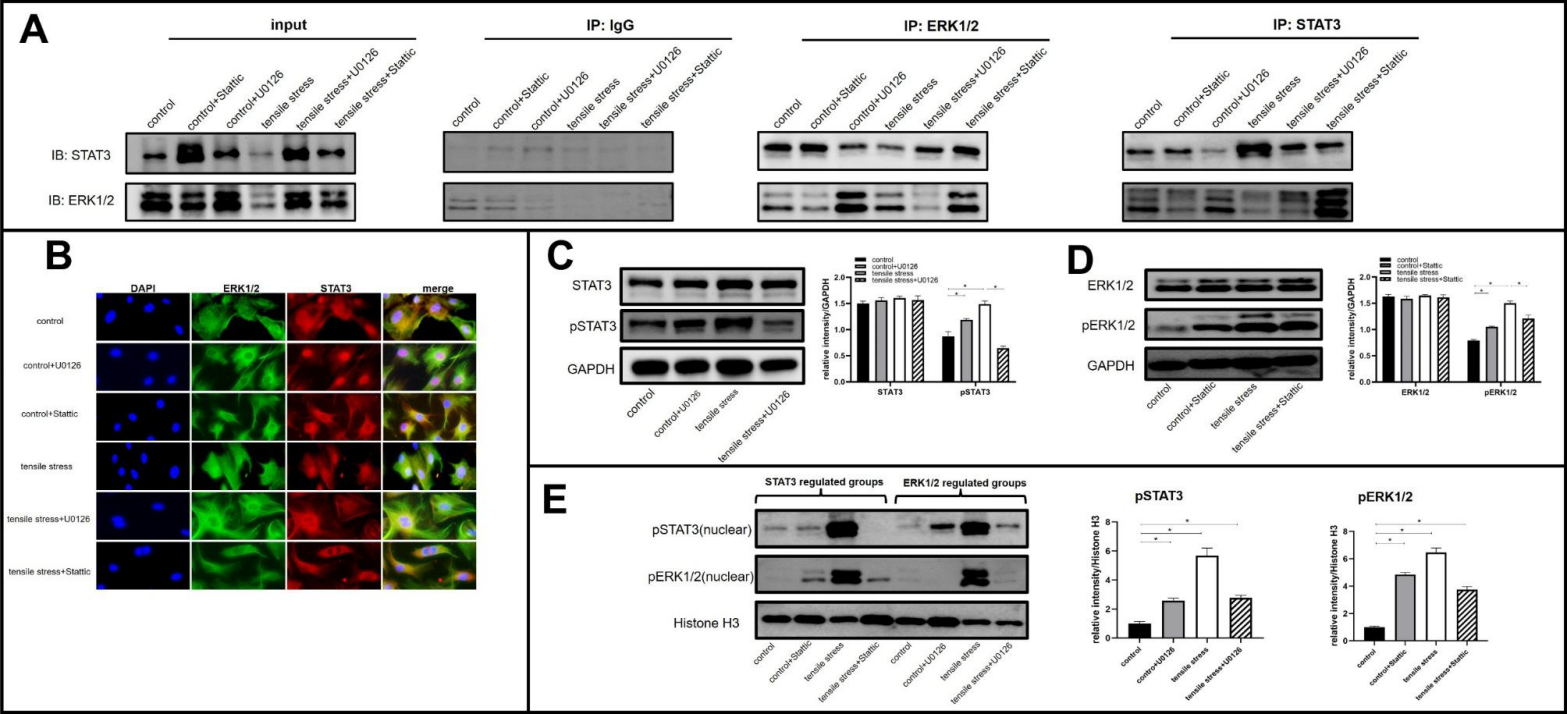
ERK1/2 and STAT3 had interaction and their activation influenced each other. (A) Representative images of Co-IP. (B) Immunofluorescence assay showed the subcellular location of ERK1/2 and STAT3 was affected by loading, U0126, and Stattic administration. (C) Western blot analysis of STAT3 and pSTAT3 protein expression in whole cell lysates. (D) Western blot analysis of EKR1/2 and pERK1/2 protein expression in whole cell lysates. (E) Western blot analysis was performed to examine the protein levels of pERK1/2 and pSTAT3 in nuclear fractions. Bars represent mean ± SD. *p < 0.05

Initially, we used Co-IP to determine whether STAT3 and ERK1/2 interacted, and the results suggested that STAT3 coimmunoprecipitated with ERK1/2 in all groups (Fig. 5A).

The subcellular localization of the two proteins was further delineated by co-immunofluorescence (Co-IF). In the control group, both ERK1/2 and STAT3 were located in the nucleus and cytoplasm. STAT3 was mostly found in the nucleus in the control + U0126 group, suggesting ERK1/2 inhibition impacted STAT3 localization. However, STAT3 inhibition did not significantly alter ERK1/2 localization in the control + Stattic group. As a result of tensile loading, STAT3 and ERK1/2 were found to accumulate near nuclear regions. In contrast, in the tensile stress + U0126 group, ERK1/2 and STAT3 were more localized in the cytoplasm. Furthermore, STAT3 inhibition decreased the loading-induced nuclear translocation of ERK1/2, as demonstrated in the in the tensile stress + Stattic group (Fig. 5B).

Western blot analysis further clarified the mutual relationship between STAT3 and ERK1/2 in a more quantitative sense. First, we detected the levels of STAT3 and pSTAT3 in whole cell lysates, finding that total STAT3 protein expression was not significantly different across groups. The control + U0126 group displayed a significantly higher level of pSTAT3 protein than the control group. However, loading-induced pSTAT3 upregulation was reduced after ERK1/2 inhibition (Fig. 5C). Our next step was to examine ERK1/2 protein changes following STAT3 inhibition. ERK1/2 expression did not differ significantly between groups. However, pERK1/2 expression was significantly higher in the control + Stattic group compared to the control group. In spite of this, Stattic administration did not impact the pERK1/2 protein expression between the tensile stress and the tensile stress + Stattic groups (Fig. 5D).

We then extracted nuclear proteins and used western blots to measure nuclear pERK1/2 and pSTAT3 expression. As shown in Fig. 5E, pERK1/2 expression was significantly higher in the control + Stattic group compared with the control group. STAT3 inhibition, however, greatly reduced loading-induced nuclear pERK1/2 expression. Regarding the impact of ERK1/2 on STAT3 expression, inhibition of ERK1/2 prohibited loading-induced nuclear pSTAT3 protein. Moreover, the control + U0126 group demonstrated a higher level of nuclear pSTAT3 protein than the control group.

## Discussion

A variety of mechanical therapies have been developed to correct craniofacial skeletal or/and dental problems, including distraction osteogenesis and orthodontic tooth movement. Molecular mechanisms by which external mechanical stimulation is translated into cells and then affects cell activities are always of interest. Currently, the purpose of our study was to examine the effect of tensile loading on calvarial osteoblasts, as well as the involvement of ERK1/2 and STAT3.

The Flexcell tension system is widely used to apply stretch forces to cell populations. The strain regimes vary depending on the type of cell population studied, and the responses induced by stretch forces vary among different types of cells. In osteoblastic cells, elongation from 0.25% [13] to 24% [14] significantly influenced survival, cytokine production, maturation, and differentiation. The upregulation of osteogenic differentiation factors was found to be dependent on strain magnitude in the region of < 18% elongation [15]. Additionally, osteoblast proliferation has been found to be stimulated by elongation between 0–12% [16], but higher levels would adversely impact it [17]. As our study focused mainly on osteoblastic function under tensile force, we exposed primary rat osteoblasts to 10% elongation as a way to enhance differentiation without compromising cell viability.

The upregulation patterns of osteogenic markers in our study were correlated with osteoblast differentiation profiling to some extent. Runx2 and Osterix are two master transcription factors that are involved at the early stage of osteoblast differentiation [18]. These two transcription factors were found to be upregulated as early as 12 h of tensile loading, which might drive the upregulation of other osteogenic markers. ALP, COL-I, and OCN are markers of mature osteoblasts [19] and in our study, they were increased at 24 h of loading. This may imply Runx2 and Osterix are the regulators to initiate loading-induced differentiation, which then triggers activation of late-stage markers such as ALP, COL-I and OCN.

It has been reported that ERK1/2 activation directly promotes the expression of Runx2 and OCN [20]. As mentioned above, Runx2 was the chief transcription factor at the early stage, while OCN was the marker of mature osteoblasts. The ERK1/2 phosphorylation in our study was sustained from 6 to 48 h of tensile loading, which suggested that ERK1/2 was involved through osteoblast differentiation process in response to mechanical loading. To further clarify its role, U0126 was used to inhibit ERK1/2. Interestingly, ALP and ARS staining revealed that ERK1/2 inhibition impaired osteoblast mineralization regardless of mechanical loading. However, ERK1/2 inhibition did not alter ALP protein expression when no mechanical loading was applied. This inconsistency could be explained by the accumulation of inhibitory effects and the presence of tensile forces. For the ALP staining test, osteoblasts were incubated in osteogenic medium supplemented with U0126 for 7 days. In contrast, the duration of U0126 treatment in the western blot was 12 h. Taken together, we concluded that tensile stress accentuated the role of ERK1/2 in osteoblast differentiation and mineralization. It is possible that ERK1/2 inhibition undermines osteoblast differentiation capacity in a time-dependent manner in a non-loading environment.

Increasing evidence suggests that STAT3 plays a role in osteogenesis depending upon cell lineages, maturation status, and external stimuli [21]. Accordingly, in our study, STAT3 exerted different effects depending upon whether loading was present or not. Inhibition of this factor reduced loading-enhanced osteogenesis-related markers and mineralization activities, but no significant interference was observed in the non-loading environment. To update, recent in vivo research [22] has shown that STAT3 signaling is involved in load-augmented bone formation. Thus, as a result of our study, STAT3 was implicated in loading-enhanced osteogenic differentiation in primary osteoblasts, but did not appear to exert a significant effect when tensile loading was not applied. In light of this discovery, we further examined whether STAT3’s lack of engagement under non-mechanical loading conditions could be attributed to complicated interactions with ERK1/2.

Advances in decoding the crosstalk between ERK1/2 and STAT3 in specific settings may provide novel insights into the mechanism behind cellular events. The inhibition of ERK1/2 in esophageal cancer activated STAT3 and the activation of STAT3 reversed the therapeutic effect of ERK1/2, resulting in drug resistance [23]. In our study, the interaction between the two pathways varied depending on the presence of tensile forces. ERK1/2 inhibition resulted in STAT3 activation in non-loading environments and vice versa. We correlated this reciprocal relationship with osteogenesis. It should be noted that ERK1/2 inhibition remarkably impeded osteoblast differentiation and mineralization in the absence of loading, despite STAT3 activation. By contrast, STAT3 inhibition did not impair osteogenesis with the compensatory activation of ERK1/2. Based on these observations, we assumed that ERK1/2 played a more prominent role in osteogenesis than STAT3 in non-mechanical loading environments. The interaction between these two pathways altered when tensile loading was applied. The activation of ERK1/2 and STAT3 by tensile loading occurred in chronological order, with ERK1/2 activation followed by STAT3 activation. Furthermore, ERK1/2 inhibition completely prevented STAT3 phosphorylation and nuclear translocation induced by loading. Although STAT3 inhibition did not impact loading-induced ERK1/2 phosphorylation (total), it disrupted phosphorylated ERK1/2 nuclear translocation. Together, STAT3 might function as a downstream mediator of ERK1/2 during mechanical loading, and STAT3 activation is essential for pERK1/2 translocation.

However, the study had certain limitations. First, late activation of STAT3 at 24 h of tensile loading may involve the synthesis of intermediate factors. Therefore, methods such as cycloheximide chase analysis could be used to further explore potential protein synthesis or degradation. Second, a more thorough elucidation of their roles in osteogenesis could be achieved by using silencing RNA to directly target ERK1/2 and STAT3 gene expression.

## Methods

### Cell isolation and culture

Murine primary osteoblasts were obtained as described in previous research [24, 25]. Briefly, the calvarial bone was carefully dissected from one-day-old neonatal Wistar rats and washed with PBS solutions containing 100 U/ml penicillin and 100 µg/ml streptomycin thrice. The semi-transparent calvarial bone was incubated with 0.25% trypsin at 37 °C for thirty minutes, then sliced and incubated with 0.1% collagenase I at 37 °C overnight. The isolated osteoblasts were collected from the final digests and resuspended in the complete medium consisting of α-MEM, 10% FBS, 100 U/ml penicillin, and 100 µg/ml streptomycin. Cells were cultured in the humidified atmosphere of 5% CO_2_.

### Mechanical stress application

Cells were seeded onto collagen I-coated six-well BioFlex® plates (Flexcell Int. Corp, Hillsborough, NC, USA) at a density of 4 × 10^5^ cells/ml. When the cells reached 80% confluence, cells were subjected to cyclic tensile stress at 10% magnitude and 0.5 Hz frequency for various durations (6, 12, 24, and 48 h). Cells not exposed to tensile stress served as the control group. After mechanical loading, cells were harvested and tested for changes in the expression of various genes and proteins [26].

### Inhibitors preparation

ERK1/2 inhibitor (U0126 solution): U0126 powder was dissolved in dimethyl sulfoxide (DMSO) to 50µM concentration as the storage solution.

STAT3 inhibitor (Stattic solution): Stattic powder was dissolved in DMSO to 20µM concentration as the storage solution.

### Real-time polymerase chain reaction (RT-PCR)

Total RNA was extracted by Trizol reagent (Invitrogen, Carlsbad, CA, USA) according to instructions. Then cDNA synthesis and subsequent amplification were performed in compliance with the manufacturer’s protocols (Takara, Tokyo, Japan). Gene-specific primers (Table 1) were designed by Shanghai Sangon Biotechnology Co., Ltd.

**Table 1:** Primer sequences and product sizes for quantitative polymerase chain reaction

Target gene	Sequences (5’-3’)^*^	Product sizes (bp)
*Runx2*	F: ATCTACACCGTGGGATATTCCA R: GCAGCATAAACGACAGGAACA	130
*Osterix*	F: CCTCTTGAGAGGAGACGGGA R: GGGCTGAAAGGTCAGCGTAT	157
*OCN*	F: GCACACCTAGCAGACACCAT R: GTGTTGTCCCTTCCCCTCTG	135
*COL-I*	F: GATGTGGAATACGAACTGGATG R: TGGGAATGCTTGTGTCTGG	134
*ALP*	F: GAACAGACCCTCCCCACGAG R: CCTGAGATTCGTCCCTCGC	104
*GAPDH*	F: ACAGCAACAGGGTGGTGGAC R: TTTGAGGGTGCAGCGAACTT	136

^*^F means forward primers; R means reverse primers

### Western blot (WB) assays

Total cellular protein (Signalway Antibody LLC, USA) and nuclear protein (Shanghai Sangon Biotechnology Co., Ltd, China) were extracted using the corresponding kit. The sample concentration was determined by a BCA protein assay kit (Beyotime, China). After being mixed with loading buffer, the protein sample was boiled at 95 °C for 5 min. The protein sample was separated by SDS polyacrylamide gel electrophoresis and transferred to PVDF membrane. Later, the blots were blocked in 5% fat-free skimmed milk for one hour at room temperature, followed by incubation with primary antibodies (Huabio, China) against Osterix (1:1000, ER1914-47), COL-I (1:1000, ET1609-68), OCN (1:1000, ER1919-20), Runx2 (1:1000, ET1612-47), ALP (1:1000, ET1601-21), ERK1/2(1:5000, ET1601-29), pERK1/2 (1:1000, ET1603-22), STAT3(1:1000, ET1607-38), pSTAT3(1:1000, ET1603-40), and GAPDH (1:1000, ET1601-4 ) overnight at 4 °C. After a three-time wash in TBST solution, membranes were probed with goat anti-rabbit IgG conjugated secondary antibody (Hubio, China) for one hour at room temperature. The blots were cut prior to hybridization with antibodies during blotting. The membranes were processed with enhanced chemiluminescence detection reagents (Applygen Technologies Inc., Beijing, China) and visualized.

### Immunofluorescence/Co-Immunofluorescence

After treatment, the cells were washed with PBS, fixed with 4% paraformaldehyde at room temperature for 30 min, penetrated with 0.5% Triton X-100 for 20 min, blocked with 0.5% BSA for one hour, and incubated with primary antibodies (Huabio, China) at 4 °C overnight. After washing thrice, cells were processed with rhodamine-labeled anti-rabbit secondary antibodies and fluorescein isothiocyanate-labeled anti-mouse secondary antibodies for one hour. Cell nuclei were stained with DAPI and images were observed with the fluorescence microscope (Leica, Germany).

### Co-immunoprecipitation (Co-IP)

The total protein was extracted as mentioned before. Co-IP was conducted as described previously [27]. 10% of the supernatants were prepared for Input, and the rest were incubated with normal IgG, anti-ERK1/2 (dilution 1:20), or anti-STAT3 (dilution 1:20) at 4 °C overnight with gentle end-over-end mixing, followed by pre-washed Protein A/G magnetic beads (Shanghai Epizyme Biomedical Technology Co., Ltd), mellow agitation, and overnight attachment. Protein A/G magnetic beads conjugates were separated by a magnetic rack. Proteins were eluted by boiling with loading buffer and then detached from beads by the magnetic rack.

### Alkaline phosphatase (ALP) staining and alizarin red (ARS) staining

The mineralization solution was prepared as previously described [28] (α-MEM containing 10% FBS, 50 µM ascorbic acid, 100 nM dexamethasone, and 10 mM β-glycerophosphate). For ALP staining, cells were incubated in mineralization media for 7 days. ALP activity was performed using an ALP staining kit (Beyotime, Shanghai, China). Briefly, cells were rinsed with PBS three times and fixed in 4% paraformaldehyde at room temperature for 15 min. They were then incubated in ALP staining solution for 15 min.

For ARS solution, cells were cultured in mineralization media for 15 days. After washing with PBS, the cells were fixed with 4% paraformaldehyde and stained with ARS solution (Sigma-Aldrich, St. Louis, MO, USA; Solarbio, Beijing, China). The quantitative analysis of Alizarin red staining images was performed by Image J software according to the standard protocols as described in our previous research [29].

### Statistical analysis

All quantitative data from three experiments were expressed as mean ± standard deviation and analyzed by GraphPad Prism (GraphPad Software, La Jolla, CA, USA). Tests for normality and homoscedasticity were performed in GraphPad Prism according to the instruction guide. Data were assessed through two way-ANOVA (for factorial designed experiments) or one-way ANOVA (for three or more groups) followed by post-hoc Tukey test. *P* < 0.05 was considered to be statistically significant.

## Electronic supplementary material

Below is the link to the electronic supplementary material.

Supplementary Material 1

## Data Availability

The datasets used and/or analyzed during the current study are available from the corresponding author on reasonable request.
