# Supplementary material for: Cyclic tensile force modifies calvarial osteoblast function via the interplay between ERK1/2 and STAT3
Source: BMC Mol Cell Biol. 2023 Mar 8;24:9. doi: 10.1186/s12860-023-00471-8 (PMC9996996; doi:10.1186/s12860-023-00471-8)

Fig. 1B

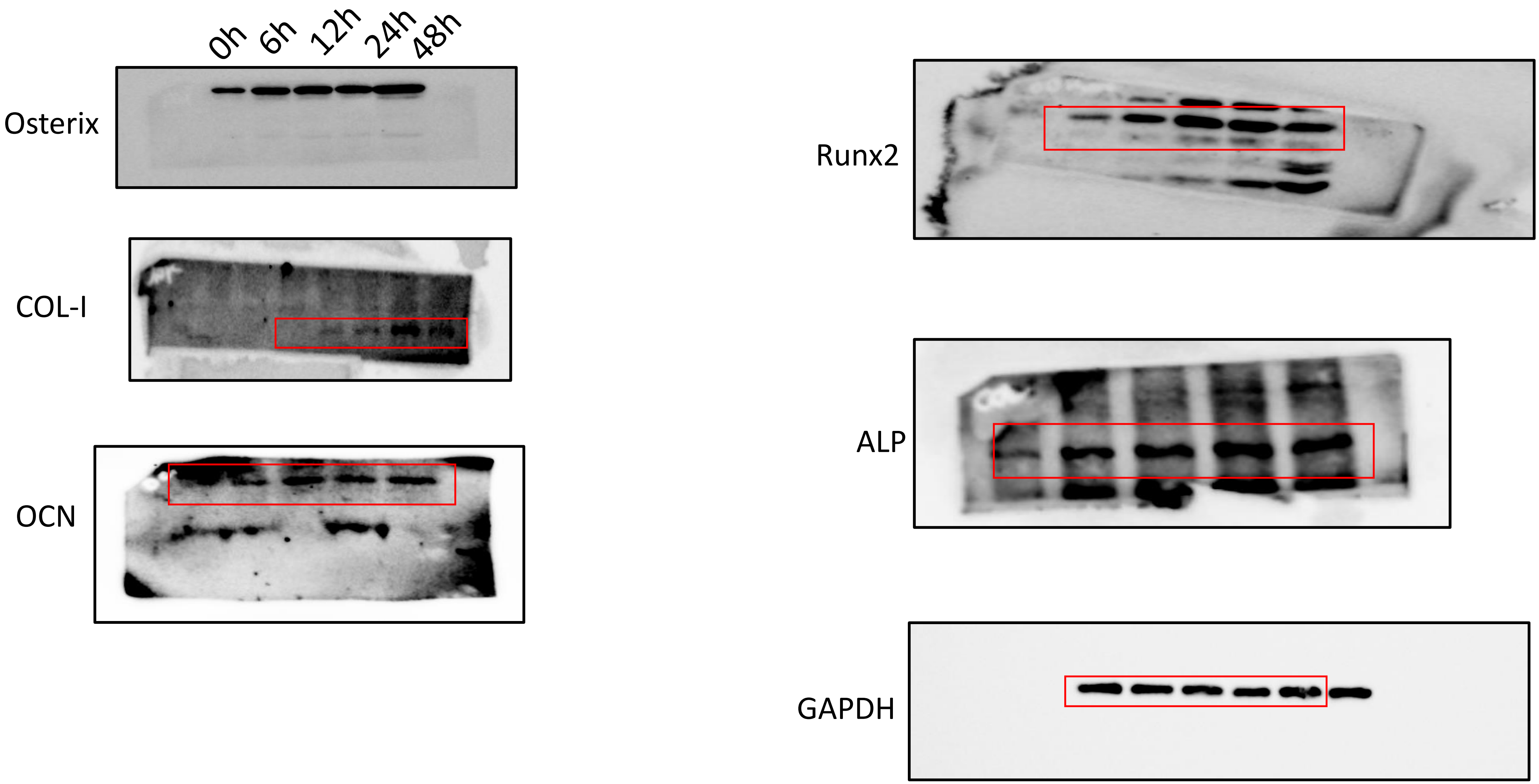

Fig. 2A

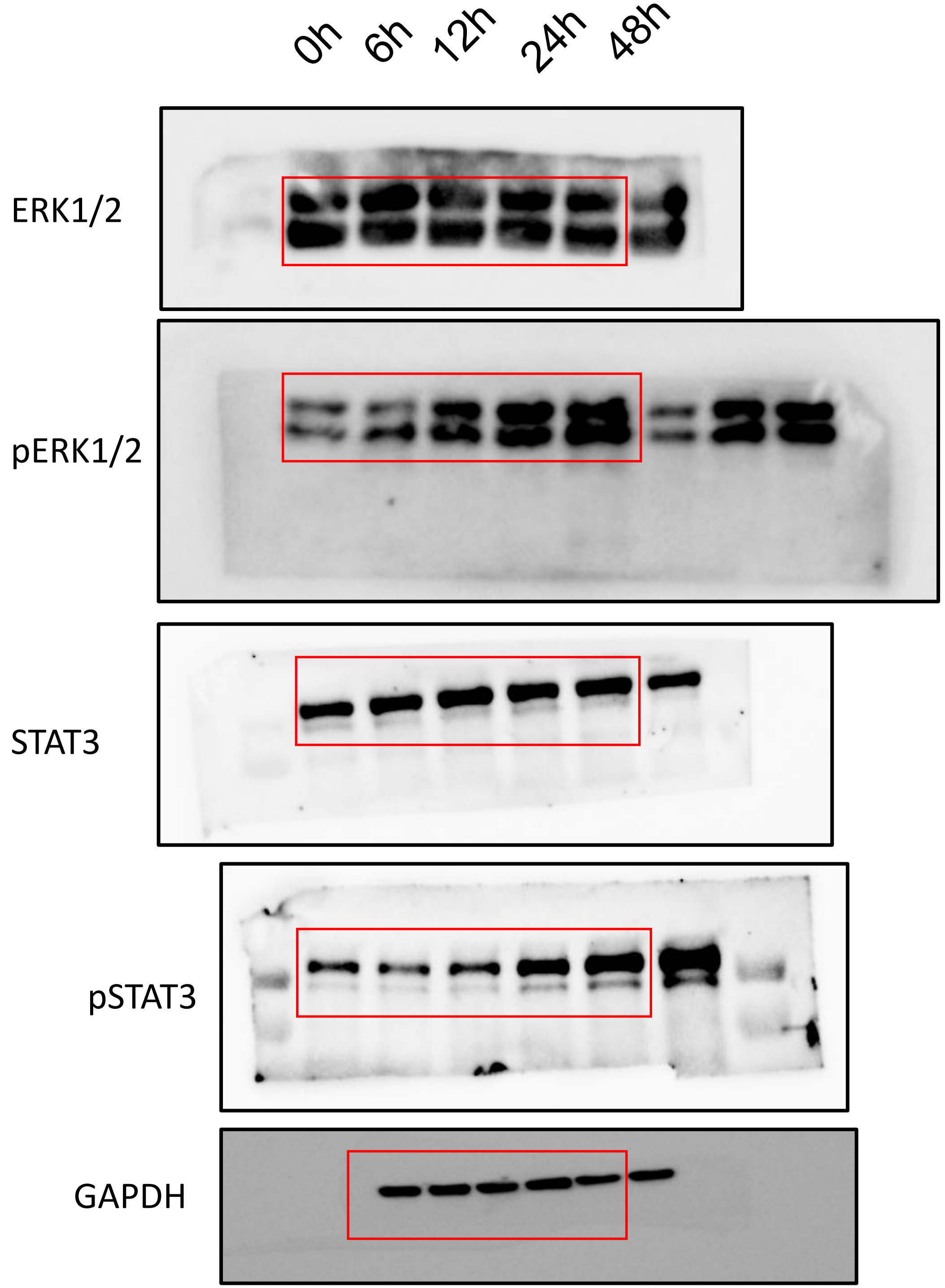

Fig. 3B

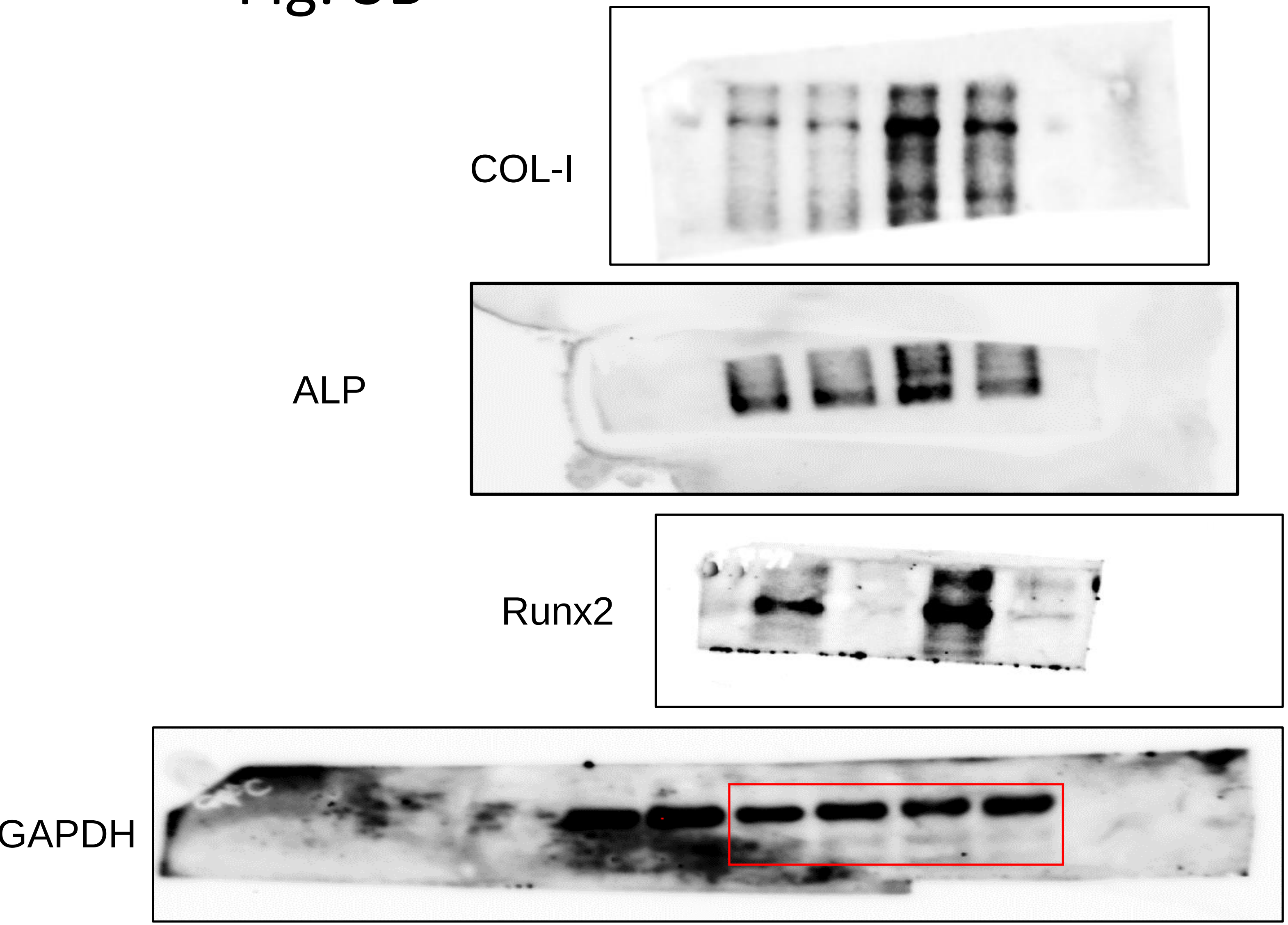

Fig. 4B

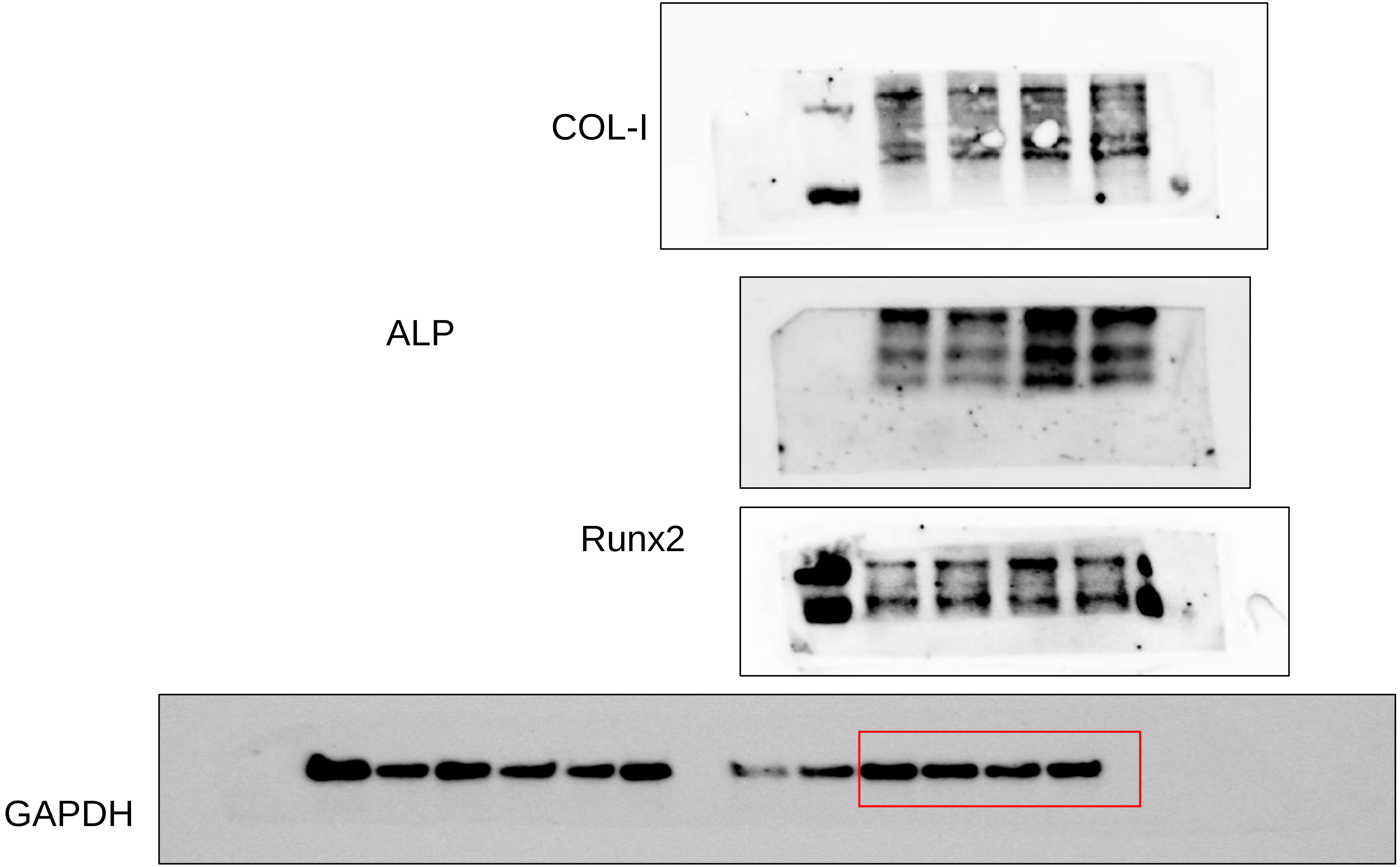

Fig. 5A

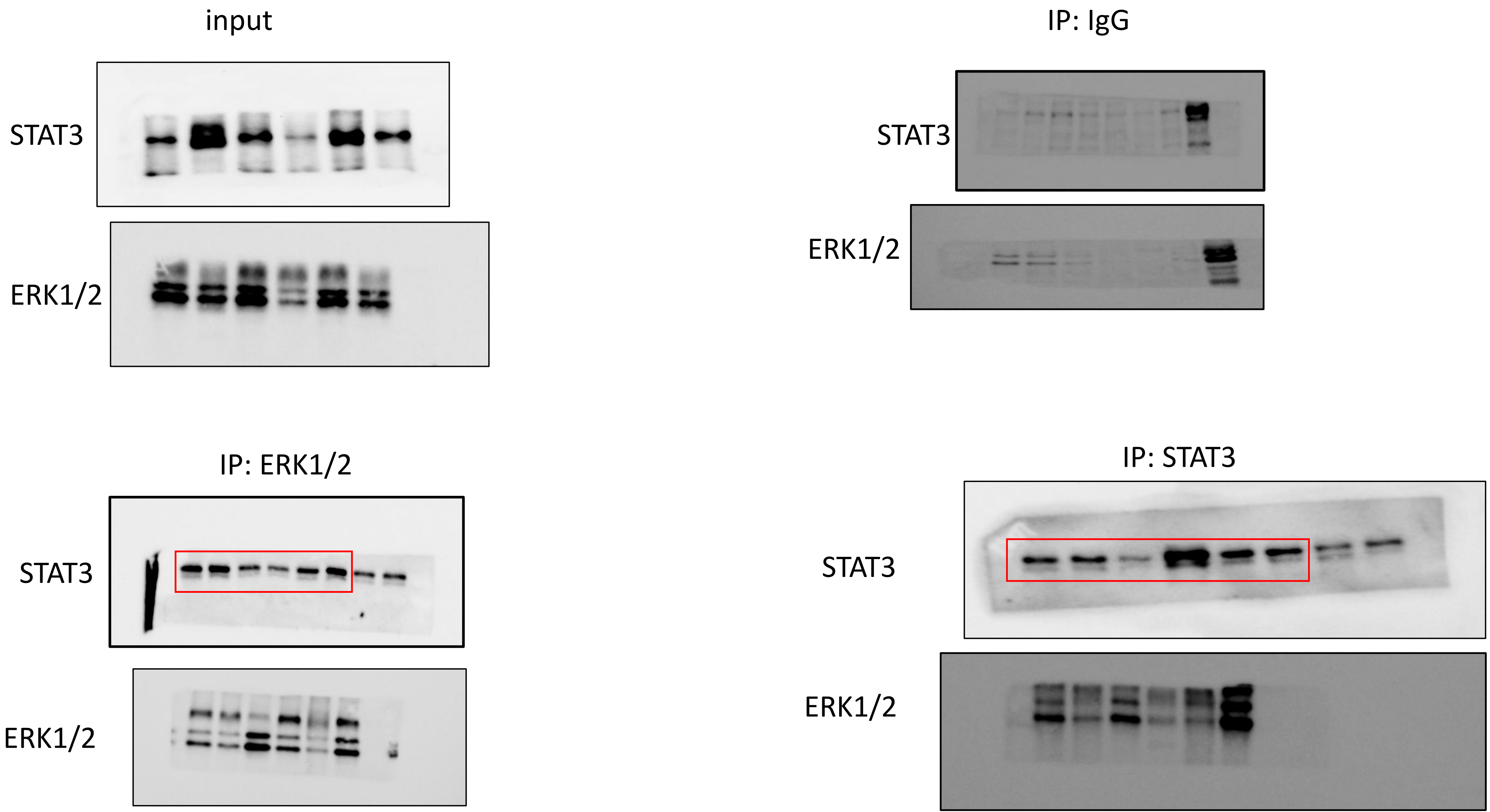
input
STAT3
ERK1/2
IP: IgG
STAT3
ERK1/2
IP: ERK1/2
STAT3
ERK1/2
IP: STAT3
STAT3
ERK1/2

Fig. 5C

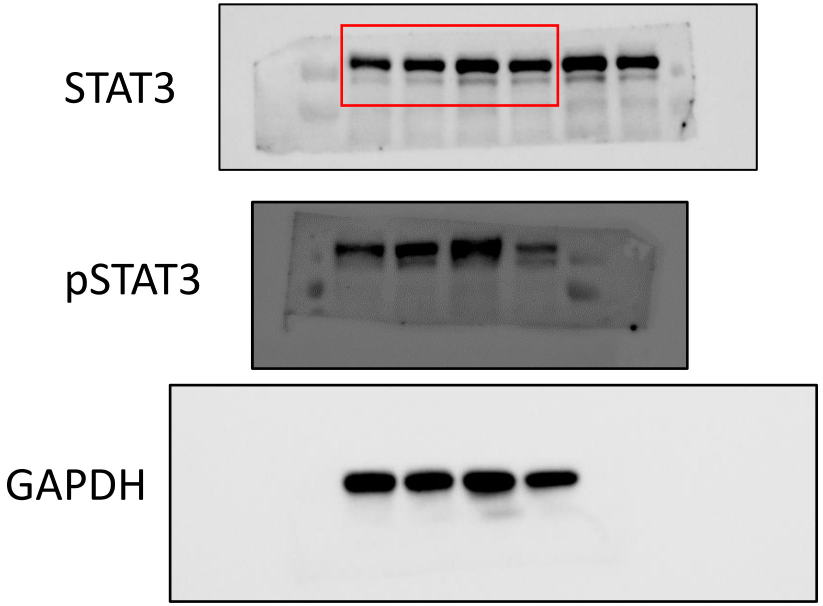

Fig. 5D

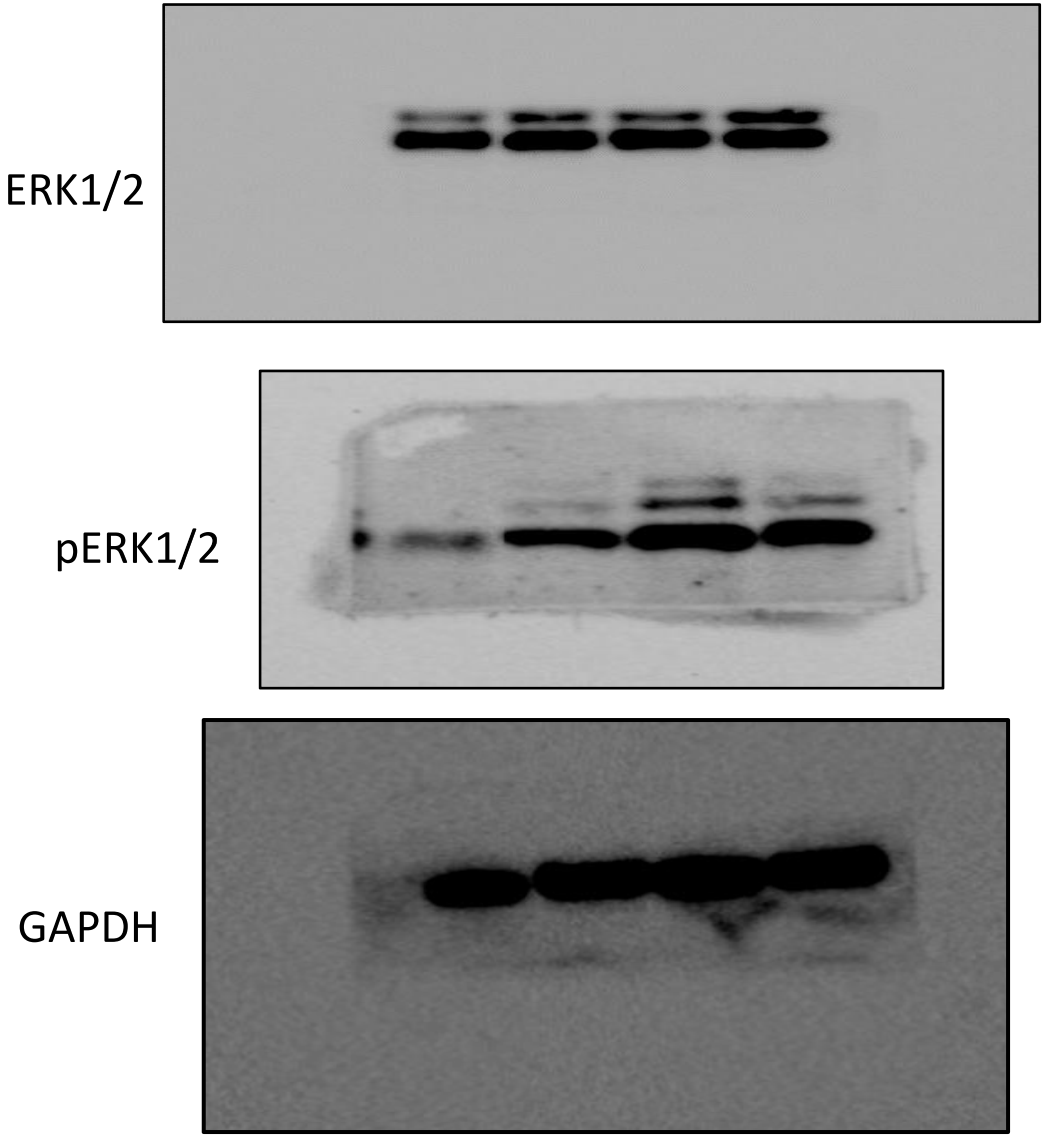

Fig. 5E

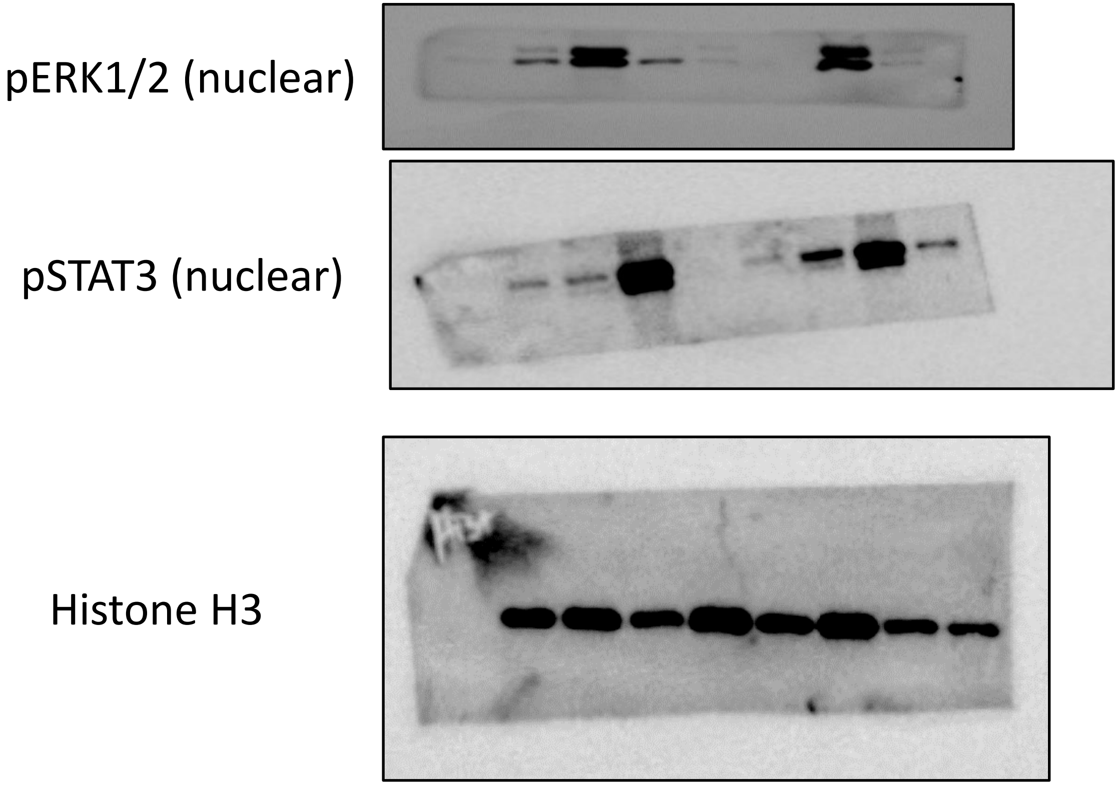

Supplement: Supplementary file 1 — Supplementary Material 1 [file 12860_2023_471_MOESM1_ESM.pdf]
